# Novel Prion Protein in BSE-affected Cattle, Switzerland

**DOI:** 10.3201/eid1801.111225

**Published:** 2012-01

**Authors:** Torsten Seuberlich, Michaela Gsponer, Cord Drögemüller, Miroslaw P. Polak, Sandra McCutcheon, Dagmar Heim, Anna Oevermann, Andreas Zurbriggen

**Affiliations:** University of Bern, Bern, Switzerland (T. Seuberlich, M. Gsponer, C. Drögemüller, A. Oevermann, A. Zurbriggen);; National Veterinary Research Institute, Pulawy, Poland (M.P. Polak);; University of Edinburgh, Edinburgh, Scotland, UK (S. McCutcheon);; Federal Veterinary Office, Liebefeld, Switzerland (D. Heim)

**Keywords:** bovine spongiform encephalopathy, BSE, cattle, prion, prion protein, Switzerland, atypical BSE, prions and related diseases

**To the Editor:** Bovine spongiform encephalopathy (BSE) is a feed-borne prion disease that affects mainly cattle but also other ruminants, felids, and humans (*1*). Currently, 3 types of BSE have been distinguished by Western immunoblot on the basis of the signature of the proteinase K–resistant fragment of the pathologic prion protein (PrP^res^): the classic type of BSE (C-BSE) and 2 so-called atypical types of BSE with higher or lower molecular masses of PrP^res^ (H-BSE and L-BSE, respectively) (*2*). C-BSE is transmitted to cattle by ingestion of contaminated meat-and-bone meal, a feed supplement produced from animal carcasses and by-products. H-BSE and L-BSE have been identified by active disease surveillance, and incidence in aged cattle is low; but little is known about their epidemiology, pathobiology, and zoonotic potential (*3*). We describe 2 recent cases of BSE in aged cattle in Switzerland in which a PrP^res^ phenotype distinct from those of C-, L- and H-BSE was unexpectedly displayed.

In April 2011, an 8-year-old cow (cow 1) died of accidental injury, with no apparent precedent clinical signs, on a farm in the canton of St. Gallen, Switzerland. In the context of active surveillance for BSE, the medulla oblongata was tested and found to be BSE positive by using the PrioStrip test (Prionics AG, Schlieren, Switzerland), a lateral-flow immunochromatographic assay for detection of PrP^res^. One month later, another cow (cow 2), 15 years of age, in the canton of Berne, Switzerland, was slaughtered because of a hind limb fracture. Information on this animal’s health status before death was unavailable. Statutory testing of the medulla oblongata gave a BSE-positive result by using the Prionics Check Western, a rapid Western blot technique (*4*). Medulla oblongata samples from the 2 animals were forwarded to the National Reference Laboratory for confirmatory testing.

In accordance with the guidelines of the World Organisation for Animal Health (*5*), BSE was confirmed for each animal by positive test results in independent, approved screening tests, of which 1 must be a Western blot (Technical Appendix). Because the tissues were severely autolyzed, target structures for the diagnosis of BSE could not be identified, and histopathologic and immunohistochemical results were inconclusive.

The Prionics Western blot detected a similar 3-band PrP^res^ glycoprofile with molecular masses of roughly 16, 20, and 25 kDa for each animal, lower than equivalent PrP protein bands detected in animals with C-BSE (Figure). Sequencing of the open reading frame of the *PRNP* gene of cow 2 (which was unsuccessful for cow 1) indicated that the encoded protein was identical to the common bovine PrP amino acid sequence (as translated from GenBank accession no. AJ298878) and therefore was not likely to account for the differences observed by Western blot testing.

**Figure:**
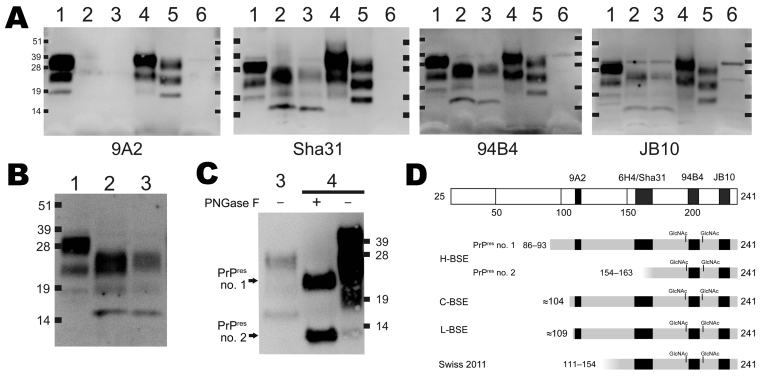
Molecular typing of the pathologic prion protein from 2 cows with bovine spongiform encepalopathy (BSE), Switzerland. Brain tissue homogenates of medulla oblongata from cows 1 and 2 and from controls with C-BSE (Switzerland), H-BSE, and L-BSE (Poland [*2*]), and a BSE-negative sample were treated with proteinase K, subjected to sodium dodecyl sulfate polyacrylamide gel electrophoresis (SDS-PAGE), and blotted onto membranes according to the protocol of the Prionics AG (Schlieren, Switzerland) Check Western test. On the basis of the antibody binding results, the N terminus of the PrP^res^ fragment in the samples from cows 1 and 2 lies between aa 111 and aa 154. Predicted PrP^res^ fragments of C-BSE, H-BSE (PrP^res^ 1 and 2), and L-BSE were adopted from the literature (*2*,*6*). A) Epitope mapping using antibodies 9A2, Sha31, 94B4, and JB10. B) Confirmatory Western blotting using antibody 6H4. C) Comparison PrP^res^ in cow 2 and H-BSE with (+) and without (–) deglycosylation with PNGaseF (antibody 94B4, SDS-PAGE with NuPAGE MES instead of NuPAGE MOPS running buffer [Invitrogen, Carlsbad, CA, USA]). PrP^res^ 1 and 2 in H-BSE samples are indicated. Molecular mass standards are shown in kiloDaltons. D) The illustration at the top represents the full-length, mature bovine prion protein and the binding sites of the antibodies used for epitope mapping (black boxes). N-terminal and C-terminal residues are indicated by numbers. PrP^res^ fragments are partially mono- and di-glycosylated, which results in the characteristic 3-band patterns in the Western immunoblot. Sites of N-linked glycosylation are shown at positions 192 and 208 (-GlcNAc). C-BSE, classic BSE; cow 1, an 8-year-old BSE-positive cow; cow 2, a 15-year-old BSE-positive cow; PrP^res^, proteinase K–resistant fragment of the prion protein; H-BSE, atypical BSE with higher molecular mass of PrP^res^; L-BSE, atypical BSE with lower molecular mass of PrP^res^. Lane 1, C-BSE; lane 2, cow 1; lane 3, cow 2; lane 4, H-BSE; lane 5, L-BSE; lane 6, negative.

We next investigated which region of the prion protein was present in these abberant PrP^res^ fragments by probing with a panel of antibodies in the Western blot that bind to different regions of the prion protein (Technical Appendix). PrP^res^ in cows 1 and 2 was readily detected by antibodies Sha31, 94B4, and JB10. By contrast, antibody 9A2, which maps to the PrP^res^ N terminus, bound only to PrP^res^ in samples from animals with C-, L- and H- BSE but not in samples from cows 1 and 2. The molecular masses of the PrP^res^ moieties from the 2 cows were also clearly distinct from those from controls with L- and H-BSE (Figure). For samples from animals with H-BSE, enzymatic deglycosylation demonstrated PrP^res^ subtypes, 1 and 2, the latter being a C-terminal PrP^res^ fragment of ≈12–14 kDa (*6*). To investigate whether the novel PrP^res^ type corresponds to PrP^res^ subtype 2, we compared samples from cow 2 with those from the H-BSE control by Western blot. The PrP^res^ type from the 2 cows reported here and PrP^res^ subtype 2 from the H-BSE control were indeed distinct (Figure).

We report a novel PrP^res^ signature in 2 cows with BSE diagnoses determined according to established criteria. Combining Western blot analysis with an epitope mapping strategy, we ascertained that these animals displayed an N terminally truncated PrP^res^ different from currently classified BSE prions (Figure). The interpretation of these findings remains difficult because neuropathologic and systematic clinical data for the 2 cases are not available. Moreover, the tissue samples were autolyzed, and the question of whether this affected the PrP^res^ molecular signature is of concern. Nonetheless, our findings raise the possibility that these cattle were affected by a prion disease not previously encountered and distinct from the known types of BSE. To confirm this possibility and to assess a potential effect on disease control and public health, in vivo transmission studies using transgenic mouse models and cattle are ongoing. Until results of these studies are available, molecular diagnostic techniques should be used so that such cases are not missed.

## Supplementary Material

Technical Appendix Details of 2 cows with bovine spongiform encephalopathy, Switzerland, 2011.
