# Supplementary material for: Novel Prion Protein in BSE-affected Cattle, Switzerland
Source: Emerg Infect Dis. 2012 Jan;18(1):158–9. doi: 10.3201/eid1801.111225 (PMC3310124; doi:10.3201/eid1801.111225)
Supplement: Technical Appendix — Details of 2 cows with bovine spongiform encephalopathy, Switzerland, 2011. [file 11-1225-Techapp_1p.pdf]

Article DOI: http://dx.doi.org/10.3201/eid1801.111225

Novel Prion Protein in BSE-affected Cattle, Switzerland

Technical Appendix

Table 1. Details of 2 cows with bovine spongiform encephalopathy, Switzerland, 2011

Case no.	Year of birth	Breed	Laboratory confirmation test results: PrioStrip [RDU]* 1st/2nd run	Laboratory confirmation test results: Check Western†
2011/1	2003	Brown Swiss	438/418	Positive
2011/2	1995	Swiss Fleckvieh	243/252	Positive

*Prionics AG, Schlieren, Switzerland. Cutoff value 90 relative density units (RDU).
†Prionics AG. Antibody used was 6H4.

Table 2. Prion protein– specific antibodies used in bovine spongiform encephalopathy testing, Switzerland, 2011*

Antibody	Epitope*	Concentration	Reference
9A2	$_{110}$WNK$_{112}$	1 µg/mL	(*1*)
6H4	$_{156}$YEDRYYREN$_{164}$	0.2 µg/mL	(*2*)
Sha31	$_{156}$YEDRYYRE$_{163}$	1:10†	(*3*)
94B4	$_{198}$HTVTTTTK$_{205}$	1 µg/mL	(*4*)
JB10	$_{230}$QRESQAYY$_{237}$	0.6 µg/mL	S. McCutcheon, unpub. data

*Amino acids and positions according to the bovine prion protein sequence (www.uniprot.org, accession no. P10279).
†Bio-Rad TeSeE Western kit (Hercules, CA, USA), concentration unknown.

References

1. Langeveld JP, Jacobs JG, Erkens JH, Bossers A, Van Zijderveld FG, van Keulen LJ. Rapid and discriminatory diagnosis of scrapie and BSE in retro-pharyngeal lymph nodes of sheep. BMC Vet Res. 2006;2:19. PubMed doi:10.1186/1746-6148-2-19

2. Korth C, Stierli B, Streit P, Moser M, Schaller O, Fischer R, et al. Prion (PrPSc)-specific epitope defined by a monoclonal antibody. Nature. 1997;390:74–7. PubMed doi:10.1038/36337

3. Feraudet C, Morel N, Simon S, Volland H, Frobert Y, Creminon C, et al. Screening of 145 anti-PrP monoclonal antibodies for their capacity to inhibit PrPSc replication in infected cells. J Biol Chem. 2005;280:11247–58. PubMed doi:10.1074/jbc.M407006200

4. Thuring CM, Erkens JH, Jacobs JG, Bossers A, van Keulen LJ, Garssen GJ, et al. Discrimination between scrapie and bovine spongiform encephalopathy in sheep by molecular size, immunoreactivity, and glycoprofile of prion protein. J Clin Microbiol. 2004;42:972–80. PubMed doi:10.1128/JCM.42.3.972-980.2004
